# Keratin 8 expression in colon cancer associates with low faecal butyrate levels

**DOI:** 10.1186/1471-230X-11-2

**Published:** 2011-01-10

**Authors:** Abdul Q Khan, Jonathan P Bury, Steven R Brown, Stuart A Riley, Bernard M Corfe

**Affiliations:** 1 Department of Oncology, University of Sheffield, Medical School, Beech Hill Road, Sheffield, S10 2JF, UK; 2 Department of Gastroenterology, Northern General Hospital, Herries Road, Sheffield, S5 7AU, UK

## Abstract

**Background:**

Butyrate has been implicated in the mechanistic basis of the prevention of colorectal cancer by dietary fibre. Numerous in vitro studies have shown that butyrate regulates cell cycle and cell death. More recently we have shown that butyrate also regulates the integrity of the intermediate filament (IF) cytoskeleton *in vitro*. These and other data suggest a link between the role of diet and the implication of a central role for the keratin 8 (K8) as guardian of the colorectal epithelium.

**Methods:**

In this cross-sectional study possible links between butyrate levels, field effects and keratin expression in cancer were addressed directly by analysing how levels of expression of the IF protein K8 in tumours, in adjacent fields and at a distant landmark site may be affected by the level of butyrate in the colon microenvironment. An immunohistochemical scoring protocol for K8 was developed and applied to samples, findings were further tested by immunoblotting.

**Results:**

Levels of K8 in colorectal tumours are lower in subjects with higher levels of faecal butyrate. Immunoblotting supported this finding.Although there were no significant relationships with butyrate on the non-tumour tissues, there was a consistent trend in all measures of extent or intensity of staining towards a reduction in expression with elevated butyrate, consistent with the inverse association in tumours.

**Conclusions:**

The data suggest that butyrate may associate with down-regulation of the expression of K8 in the cancerized colon. If further validated these findings may suggest the chemopreventive value of butyrate is limited to early stage carcinogenesis as low K8 expression is associated with a poor prognosis.

## Background

The cytoskeleton of epithelial cells includes three principal types of filaments: microfilaments, microtubules, and the intermediate filaments. Intermediate filaments are formed from heteropolymers of type I and type II keratins in epithelia, specifically keratin 8 (K8) and keratin 18 (K18) in the colon, with some expression of K7, K19, and K20. Dimerisation of keratins is mediated through pairing along their coiled-coil domains, and is regulated, at least in part, through post-translational modification of the globular domains outside these regions. Keratins provide ability to the epithelium to withstand mechanical strength as evident by the pathological phenotype seen in patients with mutations in epidermal keratins [1,2]. Analysis of the K8-null mouse revealed severe disease of gastrointestinal tract characterized by colorectal hyperplasia and inflammation [3].

Keratins not only provide mechanical strength but are also involved in various regulatory functions of the cells. Genetic knock-out experiments have revealed distinct regulatory functions of K8 and K18 [4,5]. K8 plays an important function in protecting the placental barrier function [6]. K8 and K18 may regulate cell cycle and cell growth: it is suggested that the absence of K8 or K18 disturbs the cell cycle, drives cells into the G2-S phase and leads to aberrant cytokinesis through phosphorylation of keratin and interaction with adaptor protein 14-3-3 [7]. It has been demonstrated that K8/K18 loss in mice leads to reduced hepatic size and protein synthesis [8]. K8/K18 provide resistance to apoptosis on stress and injury, and this effect may be mediated through their effect on the death receptors (DR), Fas and TNF-α. K8/K18-null mouse hepatocytes were less resistant to Fas-mediated apoptosis [9]. Various abnormalities have been described in K8 deficient mice including colonic hyperplasia [10,11], hypersensitivity of the liver to stress [3,12], and alterations in intestinal epithelial membrane proteins [13].

Human mutations in K8 or K18, the principal keratins of gastrointestinal tract, have been shown to be associated with cryptogenic cirrhosis and other liver disorders [14]. Pancreatitis has also been shown an association with K8 mutation [15]. A subset of patients with ulcerative colitis has been shown to carry missense mutations in the K8 or K18 genes. Reconstructions of these mutations in vitro (K8: G62C, 163V, K464N; K18: S230T) resulted in reduced filament assembly [16]. A proteomic analysis of morphologically normal mucosa from three group of colorectal patients: cancer, polyp and normal showed the appearance of isoforms of K8 in apparently normal mucosa in polyp and cancer patients, compared with patients with no pathology [17]. Four K8 isoforms appeared in polyp mucosa relative to normal mucosa and seven K8 isoforms appeared in cancer mucosa relative to healthy mucosa. These findings indicate alterations in K8, either at the level of expression or modification in the morphologically normal mucosa as the adenoma-carcinoma sequence progresses. Another study showing differential K8 expression in colorectal carcinoma has shown reduced expression of K8 in colorectal cancer is significantly associated with shorter patient's survival, possibly on the basis of epithelial-mesenchymal transition [18]. Taken together the data from studies of both ulcerative colitis and adenocarcinoma pathologies suggests an important role for K8 and K18 in maintenance and stability of intestinal epithelia. A characteristic of keratins is their relative stability of expression even after transformation to pathological state including transformation of normal cells into malignant cells. This property has enabled keratins to be applied as tumour markers [19].

Many epidemiological studies show an inverse relationship between dietary fibre intake and the incidence of colorectal cancer, including the large EPIC study [20]. One potential mechanism for the chemoprotective effect of fibre is through the production of short-chain fatty acids (SCFA) by fermentation in the colon lumen and regulation of epithelial homeostasis. Among the SCFAs butyrate is considered to be responsible for this chemoprotection as it has major influence on cell cycle, cell differentiation and cell death. Butyrate has been shown to induce growth inhibition and terminal differentiation in a variety of human colon cancer cell lines [21-23]. Butyrate also triggers apoptosis in various cell lines [24]. The ability of butyrate to alter various cell functions is considered to be through ability to regulate gene expression by inhibition of histone deacetylase (HDAC) [25,26]. This results in hyperacetylation of histone and enhancement of transcription factors and activation or modulation of acetylated transcription factors such as Sp1 [27], nuclear structural proteins [28], and p53 [29].

Given the importance of keratin expression and function to gastrointestinal function as evidenced by the knockout mouse and heritable predisposition studies, coupled with the ability of the chemopreventive butyrate to regulate expression and function of proteins, we sought to investigate what the effect of butyrate levels in the colon microenvironment have upon expression and localisation of K8 in the normal and neoplastic colon.

## Methods

### Recruitment and SCFA extraction

A total of 17 patients with colorectal cancer were recruited for this study, 4 patients from surgical lists and 13 from endoscopy lists [30]. Biopsies were taken from three different sites in each patient as shown in Table 1. All biopsies taken for immunohistochemistry (IHC) were fixed in formalin and embedded in paraffin. Stool samples were collected from each patient at least two weeks after endoscopic procedures, for patients recruited from Endoscopy list. Stool samples for patients recruited from surgical lists were collected before surgery. Stools were processed for SCFA extraction and butyrate concentrations determined by gas chromatography [31]. Ethics committee approval was obtained from the North Sheffield Research Ethics Committee prior to recruiting (Reference number: 06/Q2308/93). Subjects included in this study were recruited between October 2007 and June 2008.

**Table 1:** Biopsying strategy for this study

Diagnosis	Biopsy positions	Other samples
Cancer	2x Mid-sigmoid for proteomics	Stool, for short-chain fatty acid determination, including butyrate
	1x mid-sigmoid for IHC	
	1x mid-sigmoid for whole mount	Food frequency questionnaire
	2x contralateral wall (field) for proteomics	
	1x contralateral wall (field) for IHC	
	2x lesion for proteomics	
	1x lesion for IHC	

### Immunohistochemistry

All biopsies were fixed for 24 hours in formalin before paraffin embedding and cutting of serial 4-micron sections at 40-micron intervals. Antigen retrieval was performed with EDTA (1 mM, pH8) in microwave at high power for 8 mints. Avidin-biotin immunoperoxidase technique was used and diaminobenzidine was used as chromogen. Vectastain Universal RTU elite ABC kit, pk-7200 was used for blocking agent, biotinylated secondary and ABC reagent.

Sections were blocked with normal horse serum for 30 minutes at room temperature and then incubated with primary antibody (K8 mouse monoclonal M20 [32] from Abcam ab9023,, 1:200 dilution) for 60 minutes at room temperature. Sections were incubated then with Biotinylated secondary antibody (anti mouse IgG from RTU Vectastain Universal elite ABC kit, Pk-7200) for 30 minutes followed by incubation with ABC reagent for 30 minutes. PBS (phosphate buffered saline) was used for washes in between. Detection was performed using DAB kit (Vector lab- sk-4100).

### IHC Scoring for K8

Two slides containing three sections of each biopsy were stained. A maximum of 6 well-oriented crypts per biopsy, showing entire length of the crypt wall from the base abutting the muscularis mucosa through to the junction with the surface epithelium, were scored. Images were captured at 20x magnification with a Nikon D5-M camera at 2560 × 1920 resolution, stored without compression and analysed using Nikon NIS-Elements D (v 2.30) software. Scoring was performed by one observer blind to the status of the biopsy. A subset of observations was confirmed by a second independent observer.

### Protein extraction and immunoblotting for K8

Eight pinch biopsies were selected according to butyrate levels (4 high and 4 low). A modification of the intermediate filament extraction protocol described by Herman et al [33] was performed, specifically using the insoluble residue from biopsy lysis. The high-salt insoluble fraction (containing enriched intermediate filament proteins) was boiled in Laemmli buffer and separated by SDS-Page prior to immunoblotting. Primary antibody (K8 mouse monoclonal, ab9023) was used at 1:1000 concentration. The cross-reaction was visualized using HRP-conjugated secondary antibodies (Dako mouse for K8) and Western Lightning Chemiluminescence reagent plus (PerkinElmer, Boston, USA). Imaging was performed with Chemigenius Bio-Imaging system (Syngene).

### Statistical tests

Graph Pad Prism 5.02 was used for statistical analysis. Unpaired t-test was used to compare mean scores of K8 expression, including mucosal surface intensity, crypt staining intensity and crypt staining extent between mid sigmoid (MS) and contra lateral wall (CO). Unpaired t-test was also used to find relationship of two groups of butyrate with K8 expression in different parts including cancer sites. Relationship of faecal butyrate levels and K8 expression for all sites was also studied using Pearson correlation.

## Results

### Development of scoring criteria

Each biopsy from contralateral wall (CO) and mid sigmoid (MS) area was scored in three categories: surface staining intensity, crypt staining intensity relative to surface staining, and extent of crypt staining. A maximum of six crypts from six available sections on two slides were chosen for scoring from each biopsy. Means were calculated for each category scored. Intensity of surface staining was scored from 3 to 0, where 3 represented the best staining and 0 being minimal or no staining. Intensity of crypt staining was compared with intensity of surface staining and was scored 3 if better than surface staining, 2 if similar to surface staining and 1 if less than surface. Zero score was awarded if minimal or no staining observed in crypt. Score 3 was awarded if the whole extent of the crypt was stained. Score 2 being 2/3 and 1 being 1/3 of the crypt. If only few random cells were stained, that section was considered negative for scoring purposes. Immunohistochemical sections from cancer sites were scored from 0 to 3 on the basis of proportion of cells showing positive staining. Score 3 if more than 60% of cells were positively stained for K8; 2 if around half of the cells stained and 1 if less than 40% of the cells were stained with K8.

### Variation in K8 staining in normal tissue

**Figure 1:**
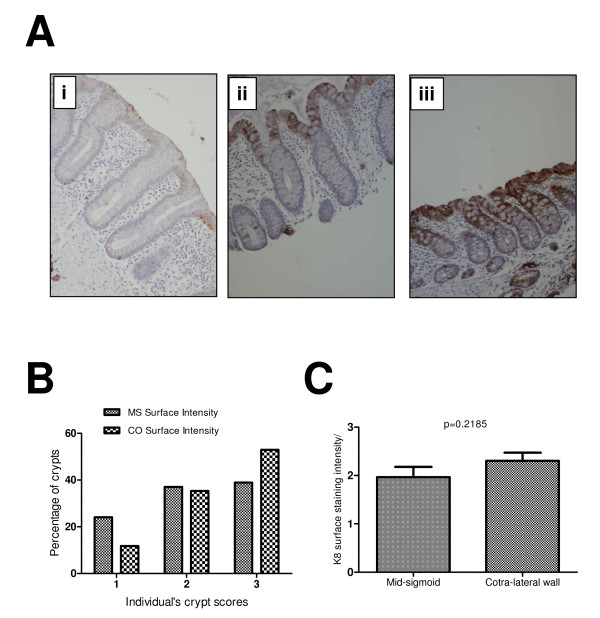
**K8 surface expression at mid-sigmoid (MS) and contra-lateral wall (CO)**. (A) Examples of various surface staining intensities. A weak staining at surface mucosa was scored 1 (Panel 1Ai) and the strong intensity was scored as 2 (Panel 1Aii). (B) Most common pattern both at MS and CO was 3 suggesting that K8 expression is most strongly expressed at the mucosal surface (38.88% at MS and 52.94% at CO). (C) Unpaired t-test shows a high mean value (2.3) at CO compared with MS (1.96) indicating a trend towards a higher surface expression of K8 around cancer compared to the distant mucosa.

K8 staining was observed as diffusely distributed in the cytoplasm of the epithelial cells (Figure 1A). Expression of K8 was noted to varying degrees along the crypt villus axis, and with varying intensities in the crypt and at the mucosal surface. In total, immunohistochemical data was available for 17 patients. Fifteen patients had good sections for scoring purposes from both (MS and CO) sites.

K8 mucosal surface staining intensity was not uniform in all sections. Examples of various staining intensities at mucosal surface are shown in Figure 1A. Figure 1, Panel Ai shows a weak staining intensity of K8 at the surface mucosa (score1) compared to Panel Aii (score2) and Panel Aiii which shows the highest staining intensity (score3). Although there was variation of staining intensity among biopsies from different patient at both sites, there was consistency among the sections from the same biopsy. When staining intensities of crypts (at surface mucosa) were compared between the landmark and the contralateral sites, 52% crypts shows strong staining intensity compared to 38% at the landmark site, with correspondingly fewer sites showing weak staining (Figure 1B). These data indicate a trend towards an increase in staining intensity at surface mucosa during progression towards the cancer from normal mucosa of the mid-sigmoid. This is also evident from higher mean score (2.3) at CO compared to MS (1.96) score (Figure 1C).

**Figure 2:**
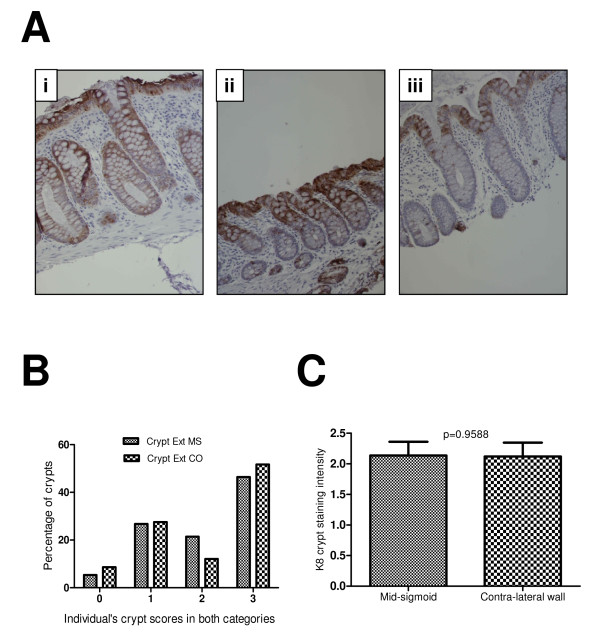
**K8 expression along the crypt axis**. Figure 2Ai shows K8 staining reaching to the bottom of the crypt, but in some crypts it reached only the upper one third (Aii) and in others upper two third was stained with K8 (A-iii). The relative occurrence of each class of staining is show in Figure 2B. Only few percent of crypts were without staining (5.37% in MS group and 8.62% in CO group) but majority of the crypts scored K8 staining reached to the bottom in both MS and CO (51.72% in CO and 46.24% in MS). There was no significant difference in the mean scores in two groups (C).

Examples of differing degrees of staining along the crypt-villus axis are shown in Figure 2A. Figure 2A.i shows an example of staining reaching the bottom third of the crypt, whereas Figure 2A.iii shows an example of K8 expression with poor crypt penetrance. Intermediate examples, as shown in Figure 2A.ii, were also observed. There was general within-subject consistency in terms of staining pattern. The relative occurrence of each class of staining extent is shown in Figure 2B. Most common pattern seen both at MS (46.24%) and CO (53.57%) was 3, showing that staining reached up to lower third of the crypt axis. In contrast to the findings for staining intensity, there was not a marked difference in the staining extent patterns between landmark and contralateral sites. A mean score of less than 2 (1.75 at MS and 1.66 at CO) was observed for intensity of the crypt staining (Table 2) implicating the stronger mucosal staining compared to the intensity of crypt staining.

**Table 2:** Summary table of differences in staining between mucosal sites

	Mid-sigmoid (mean)	Contralateral Wall (mean)	P value
Surface staining intensity	1.96	2.31	0.219
Crypt staining intensity	1.75	1.66	0.614

Extent of crypt staining	2.13	2.12	0.959

### K8 patterns and scoring in cancer tissue

**Figure 3:**
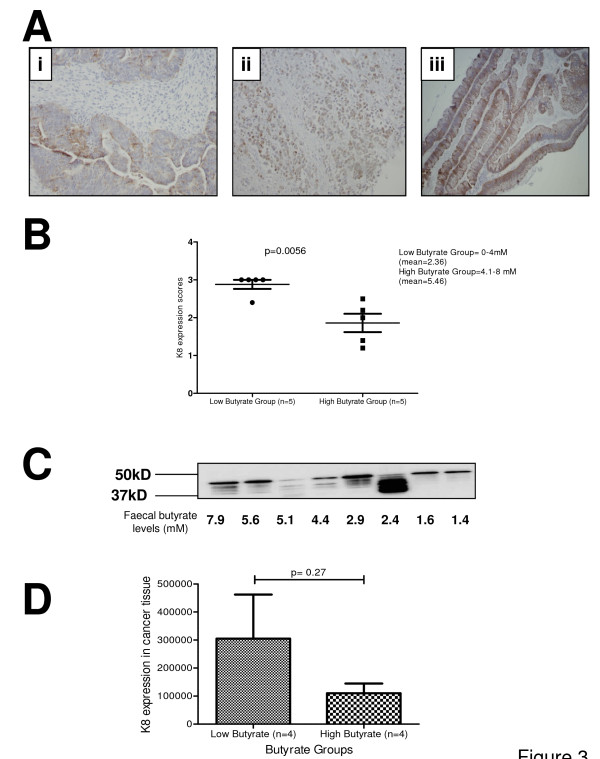
**K8 expression in cancer tissue**. Panels Ai-iii shows various examples of K8 expression scores in cancer tissue. Panel B: A significant difference of K8 expression in cancer tissue was observed between high (mean = 5.46 mM) and low butyrate (mean = 2.36 mM) groups (p = 0.0056). Panel C shows immunoblots for K8 in cancer tissue: a total 8 samples were used, 4 with high faecal butyrate levels and 4 with low faecal butyrate levels. Panel D: Densitometry result shows an inverse relationship between faecal butyrate levels and K8 expression in the cancer tissue.

Biopsies from 14 patients' cancer tissues were stained for K8. There was a wide variation in staining patterns probably because of regional heterogeneity of the tissue. Only two samples showed a score of a less than 2 i.e. most cancer tissues showed at least 50% expression of K8. None of the sample was negative for K8 which is consistent with previous results [34]. It was noted that highly undifferentiated tumours have a very patchy distribution of K8 (Figure 3-Aii) whereas more differentiated tumours have a pattern where cells along the mucosal surface were more positive for K8 and deep cells were either negative or showed less K8 expression (Figure 3A-iii).

### Relationship between faecal butyrate levels and K8 expression in morphologically normal mucosa

Faecal butyrate scores were available for 9 patients. Patients were split into haptiles by butyrate level (Low butyrate group: 0-4 mM, mean 2.36; High butyrate group >4 mM, mean 5.54). At the morphologically normal sites there was no statistically significant difference in K8 expression between two butyrate groups in any of the three categories of scoring (Table 3) both at either mid sigmoid and field. However it was noted that all scores showed a decrease from low to high butyrate, indicating a consistent pattern or trend towards decreased expression of K8 with elevated butyrate. Pearson correlation coefficient (Table 4) also did not reveal any significant relationship between faecal butyrate levels and K8 expression in any scored categories at all three sites.

**Table 3:** Summary table of differences in staining at mucosal sites by differing butyrate level

Tissue type	Keratin expression score (mean)		P value
		
	Low butyrate (n = 5, mean = 2.4 mM)	High butyrate group (n = 4, mean = 5.5 mM)	
MS surface intensity	2.12	1.4	0.12
MS crypt intensity	1.84	1.66	0.6
MS crypt extent	2.24	1.76	0.44
CO surface intensity	2.44	2.15	0.58
CO crypt intensity	1.82	1.67	0.59
CO crypt extent	2.12	2.0	0.85

**Table 4:** Summary table of Pearson correlation coefficient between faecal butyrate levels and K8 expression at mid-sigmoid, contralateral wall, and carcinoma

	N	Correlation coefficient	P value
Surface staining at MS	9	-0.209	0.59
Surfance staining at CO	9	-0.087	0.82
Crypt intensity staining at MS	9	-0.069	0.86
Crypt intensity staining at CO	9	-0.145	0.71
Extent of crypt staining at MS	9	-0.050	0.89
Extent of crypt staining at CO	9	0.014	0.97

Staining in cancer tissue	10	0.050	0.15

Pearson correlation coefficient between faecal butyrate levels and K8 expression at mid-sigmoid, contralateral wall and carcinoma

### High butyrate levels are associated with low K8 expression in cancer tissue

Patient data was split into haptiles for butyrate level as described above. Low and high butyrate groups were compared for K8 expression (Figure 3B). A highly significant difference (p = 0.0056) was observed in two groups with higher butyrate levels associated with a significant decrease in K8 staining. Intermediate filament proteins were extracted from duplicate samples and analysed for level of expression by immunoblotting. All cross-reacting bands were quantified for the scoring purposes (Figure 3C). Cross-reactions were quantitated from subjects' samples for higher and lower haptile by butyrate concentration. These results also suggested a similar inverse relationship between faecal butyrate levels and K8 expression in cancer tissue (Figure 3C &3D) although the considerable variation between samples resulted in a non-significant finding.

## Discussion

There is much emphasis on early detection of colorectal cancer and for that reason knowledge of various protein alterations which occur during carcinogenesis is important. K8 is one of major keratins in the colorectal mucosa. Through our scoring system we tried to investigate any progressive change in K8 expression from distant mucosa (MS in our study) to the cancer's field (CO) to the cancer. There was no significant difference in crypt staining intensity and crypt staining extent but there was a clear trend towards higher K8 expression in the cancer's field when compared to the distant mucosa (MS) (Figure 1C). Polley et al. [17], using a proteomic approach, also showed that there is an increase in expression of some K8 isoforms in peri-adenomatous fields. Polley et al. were unable to identify the isoform, but there is consilience insofar as both independent studies find an increase in keratin 8 with cancer progression, including in the fields around cancer.

Our scoring system for K8 expression was used to study possible relationships between the faecal butyrate (as a proxy measure of lumenal butyrate), keratin expression and colorectal cancer. We hypothesized that K8 may be altered in colonic mucosa in response to butyrate. Development of a scoring system in three categories including mucosal surface staining intensity, crypt staining intensity, and extent of crypt staining allowed testing of this hypothesis. Expression of K8 in tumours was associated with butyrate level. These data were supported by western blot analysis using a K8-specific antibody to immunoprobe the insoluble protein (K8-rich) fraction after biopsy lysis. We noted the appearance of faster migrating forms on this blot. This could be attributed to an altered profile of phosphorylation, which has been shown to link with cancer progression in the colon [35], although phosphorylation at S73 and S431 was not associated with an alteration in protein mobility in gels, K8 is a highly modified molecule. It is our working hypothesis that these bands represent proteolytically cleaved forms associated with the cancer degradome [36].

Taken alone the reduction in keratin 8 expression associated with butyrate would be seen as a risk factor for tumour progression [18], although data would need to be interpreted against a wider panel protein alterations to establish whether the global risk was actually altered. A reduction in keratin expression is observed during epithelial to mesenchymal transition, where epithelial cytoskeletal proteins are replaced with proteins more commonly associated with mesenchymal cells such as vimentin. Although not particularly widely described in colorectal oncology, progression through EMT is associated with a poor prognosis. There are precedents for nutrients associated with chemoprotective effects actually to elevate risk in the cancerized colon, notably folate [37].

One possible limitation of the study is the use of a single sampling point for faecal butyrate. A recent report highlighted the levels of intraindividual variation in SCFA concentration and SCFA production are labile, though it did suggest that concentrations were more stable as a measure than total output [38]. We reason that cells in the epithelium are exposed to a concentration, not a production rate, and have used this measure. We have found that other markers strongly correlate with faecal butyrate [39] suggesting relationships can be found, and that by implication the relationship between faecal butyrate and keratin expression is, at best, weak. A further hypothesis which may account for these findings could be that butyrate is a proxy measure of an unidentified bioactive secondary metabolite which is modulating keratin expression, or that direct interaction between butyrate-producing endosymbionts and the mucosa mediates this effect.

There was a consistent trend of increase in K8 expression in the morphologically normal tissues that did not reach significance. This observation merits further study to distinguish any possible causal role for K8 in cancer progression and the potential of manipulation of the colon luminal environment as a chemopreventive strategy.

## Conclusions

The pattern of K8 expression in normal and cancer mucosa was reported by Fujiski [34]. Our data are consistent with their findings and extend their findings by providing a scoring system, by analysing the potential relationship between K8 staining and butyrate, and by cross-validating the data with a second independent methodology. Our data suggest there may be an increase in K8 expression from landmark to contralateral fields (supporting the findings of Polley et al [17]). We also suggest that there may be a very weak trend for butyrate to reduce expression of K8 in morphologically normal tissue, but that this relationship is stronger in tumour tissue.

Further studies could compare K8 expression in apparently normal mucosa from normal screened population and cancer patients, to establish whether there is any progressive and identifiable change in K8 expression during carcinogenesis.

## Competing interests

The authors declare that they have no competing interests.

## Authors' contributions

AQK developed the staining method, recruited subjects, undertook all staining, scoring, western blotting and wrote the first draft of the paper; JPB supervised the development of staining and scoring protocols, undertook second scoring and supervised statistical analysis; SRB supervised and undertook surgical sampling, co-supervised the project with BMC and edited the draft manuscripts; SAR took overall clinical responsibility for the overarching study, undertook endoscopic sampling and edited the draft manuscripts; BMC conceived and directed the study and produced the final version of the manuscript. All authors read an approved the manuscript.

## Pre-publication history

The pre-publication history for this paper can be accessed here:

http://www.biomedcentral.com/1471-230X/11/2/prepub
